# Recent Advances in the Nickel-Catalyzed Alkylation of C-H Bonds

**DOI:** 10.3390/molecules29091917

**Published:** 2024-04-23

**Authors:** Franc Požgan, Uroš Grošelj, Jurij Svete, Bogdan Štefane, Hamad H. Al Mamari

**Affiliations:** 1 Faculty of Chemistry and Chemical Technology, University of Ljubljana, Večna pot 113, SI-1000 Ljubljana, Slovenia; franc.pozgan@fkkt.uni-lj.si (F.P.); uros.groselj@fkkt.uni-lj.si (U.G.); jurij.svete@fkkt.uni-lj.si (J.S.); bogdan.stefane@fkkt.uni-lj.si (B.Š.); 2 Department of Chemistry, College of Science, Sultan Qaboos University, Muscat 123, Oman

**Keywords:** C-H bond functionalization, C-H bond alkylation, Ni-catalysis, chelation assistance, cyclometalation

## Abstract

Functionalization of C-H bonds has emerged as a powerful strategy for converting inert, nonfunctional C-H bonds into their reactive counterparts. A wide range of C-H bond functionalization reactions has become possible by the catalysis of metals, typically from the second row of transition metals. First-row transition metals can also catalyze C-H functionalization, and they have the merits of greater earth-abundance, lower cost and better environmental friendliness in comparison to their second-row counterparts. C-H bond alkylation is a particularly important C-H functionalization reaction due to its chemical significance and its applications in natural product synthesis. This review covers Ni-catalyzed C-H bond alkylation reactions using alkyl halides and olefins as alkyl sources.

## 1. Introduction

Functionalization of C-H bonds has emerged as a powerful strategy for converting inert, nonfunctional C-H bonds into their corresponding reactive counterparts [1,2,3,4,5,6,7]. Thus kindly delete the last part of original statement which is highlighted beginning with “C-Metal…..reagents. Since C-H bonds are ubiquitous in organic molecules, controlling which C-H bond to cleave and functionalize is a formidable challenge. The problem of regioselectivity, or site-selectivity, has been partially addressed using monodentate or bidentate directing groups [8,9,10]. Such groups are Lewis bases and can interact with the Lewis acidic metal by bringing it in proximity to a C-H bond to be cleaved. Thus, transition-metal catalyzed, selective activation of C-H bonds, followed by their functionalization to directly form valuable C-C or C–X bonds (X = N, O or S), has revolutionized organic synthesis by enabling the assembly of complex molecular structures from simple precursors. In contrast to conventional cross-coupling reactions (e.g., Suzuki, Negishi or Stille) [11,12,13], C-H bond functionalization does not require the use of pre-functionalized starting materials, such as organometallic reagents, thus reducing the number of synthetic steps and the amount of waste. From this point of view, C-H bond functionalization contributes to more economically and ecologically compatible synthesis protocols, and thus also follows the principles of green chemistry [14]. Successful direct functionalization of C-H bonds was initially achieved using powerful catalysts, typically second-row transition metals such as Pd- [15,16,17,18], Rh- [19,20,21,22,23] or Ru-complexes [24,25,26,27,28], which tolerate a wide range of functionalities and common oxidants and are often stable in air and water [29,30]. Despite their great success in organic synthesis, the low abundance and high price or toxicity of some of these metals necessitated the development of new C-H bond functionalization protocols using non-noble metals. Thus, the strategy was extended to more earth-abundant first-row transition metals [31], such as Ni [32,33,34], Mn [35,36], Fe [37,38] and Co [39,40,41,42,43]. Nickel appears to be an adequate replacement for palladium, as it lies in the same group of the periodic table and can often perform the same reactions as palladium; more importantly, its use in organic synthesis could also lead to the discovery of novel reactions [44]. Various nickel oxidation states, from Ni(0) to Ni(IV), allow for different redox pathways in the catalytic cycle of a given reaction. In fact, nickel catalysts have been widely used for reactions involving alkenes and alkynes, such as oligomerization or cross-couplings [45,46]. While progress in the Ni-catalyzed activation/functionalization of C-H bonds has only recently been advanced to a synthetic level comparable to the direct functionalization of C-H bonds catalyzed by late or noble transition metals, Kleiman and Dubeck reported the formation of a nickelacycle from the stoichiometric reactions of azobenzene and NiCp_2_ complexes as early as 1963 [47]. However, the catalytic functionalization of C-H bonds by nickel complexes was initially limited to acidic C-H bonds in certain aromatic systems, and there was a lack of general and reliable methods for the activation of non-acidic C-H bonds in various organic substrates, such as azoles [48]. There is no doubt that the alkylation reaction, i.e., the coupling between an alkylating agent and a nucleophile, is an important C-C bond-forming reaction and has been widely applied in organic synthesis. In this review, we focus on the alkylation reactions of C-H bonds mediated by nickel compounds as applied to a variety of organic substrates that can be found in the literature to date, while other nickel-catalyzed C-H bond functionalizations have been reviewed elsewhere [49,50]. 

There are other modern and significant strategies for C(sp^3^)–H functionalization. Dearomatization by the hydride transfer strategy has emerged as a powerful method for achieving the ultimate goal of C-H bond functionalization [51]. The hydride transfer strategy has enabled access to various complex heterocycles through a cascade of reactions involving dearomatization and [5+1] and [5+2] cyclizations [52]. In addition, functionalization of C-H bonds has been achieved by the 1,5-hydrogen atom transfer (1,5-HAT) strategy [53]. 

Although the use of nickel in the direct functionalization of C-H bonds is an attractive area of research, and could certainly compete with other metal-catalyzed reactions in the future, synthetic methods for nickel-catalyzed C-H bond alkylation are rather scarce.

A related review covering Ni-catalyzed C-H bond functionalization reactions was reported [54]. The recent review specifically addressed intramolecular and intermolecular Ni-catalyzed functionalization reactions towards synthesis of N-containing heterocycles in the period 2008–2021 [54].

**Scheme 1:**
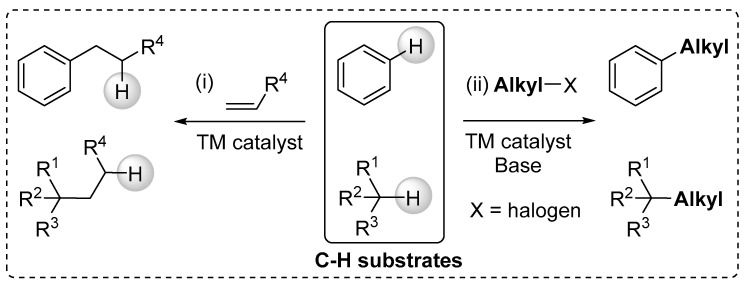
Two representative transition-metal-catalyzed C-H bond alkylation strategies.

In this review, emphasis is placed on the alkylation reactions of C-H bonds mediated by nickel compounds as applied to a variety of organic substrates that can be found in the literature to date (2004–2022). The review covers the following two main strategies for transition metal-catalyzed C-H bond alkylation: either (i) using alkenes as alkylating agents by adding the substrate C-H bond across the C=C double bond of the alkene, or (ii) introducing an alkyl moiety by using alkyl halides (or their relatives) as coupling partners, the first strategy being more economical and generating less waste (e.g., no base required) (Scheme 1). 

The present review covers methods for Ni-catalyzed C-H bond alkylation reactions to various carbocyclic and heterocyclic substrates with alkyl halides and olefins. In addition, the review coverage includes the use of directed Ni-catalyzed C-H bond alkylation reactions by chelation assistance using directing groups. 

## 2. Discussion 

### 2.1. C-H Bond Alkylation with Alkenes

**Scheme 2:**
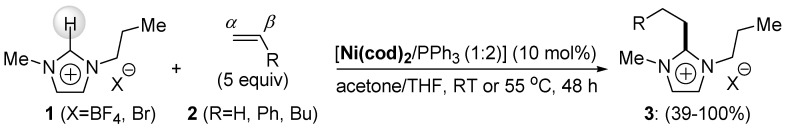
C-H bond alkylation of imidazolium salts.

In 2004, Cavell et al. reported a seminal work on Ni(0)-catalyzed C-H bond functionalization in which imidazolium salts **1** were successfully 2-C-alkylated with ethylene and other 1-alkenes **2** in the presence of the Ni(cod)_2_/PPh_3_ catalytic system, leading predominantly to linear products **3**, in which a C-C bond was formed between the *α*-carbon of the 1-alkene and the 2-C of substrate **1** (Scheme 2) [55]. The reactivity of 1-alkenes when coupled with imidazolium salts seems to be determined by steric elements, since ethylene (1 bar) was more reactive than 1-hexene. Both ionic liquids, tetrafluoroborate and bromide, were equally reactive with the alkenes used in this study.

**Scheme 3:**
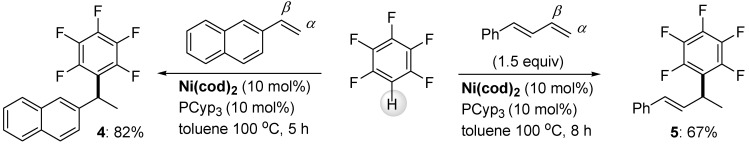
Activation of a C-H bond in pentafluorobenzene.

Later, the same Ni(0) catalyst precursor, but using PCyp_3_ as a ligand, was used for the alkylation of pentafluorobenzene with alkenes (2-vinylnaphthalene and (*E*)-buta-1,3-dien-1-ylbenzene), as shown by Hiyama and coworkers (Scheme 3) [56]. A significant influence of the ligands on the yield of the reaction was observed, as PMe_3_, PBu_3_ or P*^t^*Bu_3_ ligands were much less effective and gave only trace amounts of coupling products. In contrast to Cavell’s work (*vide supra*), the alkene in this particular reaction reacted preferentially at the *β*-carbon atom with pentafluorobenezene, leading to the branched products **4** and **5**. Although only two examples of alkylation reactions were presented in Hiyama’s paper, it was clearly shown that, under the reaction conditions used, the insertion of Ni(0) into a C-H bond was favored over insertion into a C-F bond. This was in sharp contrast to previous studies regarding the oxidative addition of Ni(0) to polyfluorobenzenes in which the C-F bond had reacted preferentially [57,58].

**Scheme 4:**
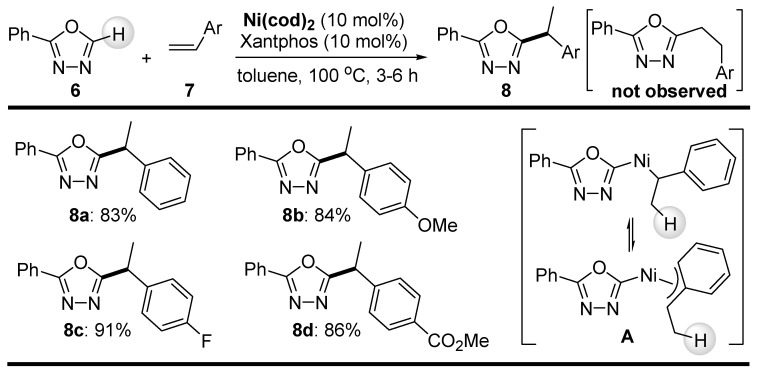
Formation of branched 5-alkyl-2-phenyl-1,3,4-oxadiazoles.

Considering the high acidity of the 5-C-H bond of 2-phenyl-1,3,4-oxadiazole (**6**), this bond was regioselectively activated by using a catalytic amount of a Ni(cod)_2_ complex, together with phosphine ligands, coupled with styrenes **7** to give branched alkylated oxadiazoles **8** (Scheme 4) [59]. The bite angle of the phosphine ligand appeared to play an important role in the reaction outcome, and the Xantphos ligand was found to dramatically increase the yield of the oxadiazole products **8**. For comparison, oxadiazole **6** gave with styrene only 17% of the corresponding product in the presence of PCy_3_ ligand, while the Xantphos ligand gave 83% of the same product under otherwise identical reaction conditions. Styrenes containing either an electron-withdrawing or an electron-donating group were comparably reactive in this coupling reaction, whereas simple aliphatic alkenes and acrylates did not yield the corresponding alkylated 1,3,4-oxadiazole products. The importance of this nickel-catalyzed alkylation with styrenes was recognized by the observed regioselectivity, which is in a stark contrast to the similar ruthenium-catalyzed reactions that yield predominantly linear coupling products [60,61]. The observed branch selectivity with styrenes in this nickel-catalyzed reaction could be explained by the preferential formation of the π-benzyl nickel intermediate **A**, which is not possible with simple alkenes and acrylate esters.

### 2.2. C-H Bond Alkylation with Alkyl Halides

**Scheme 5:**
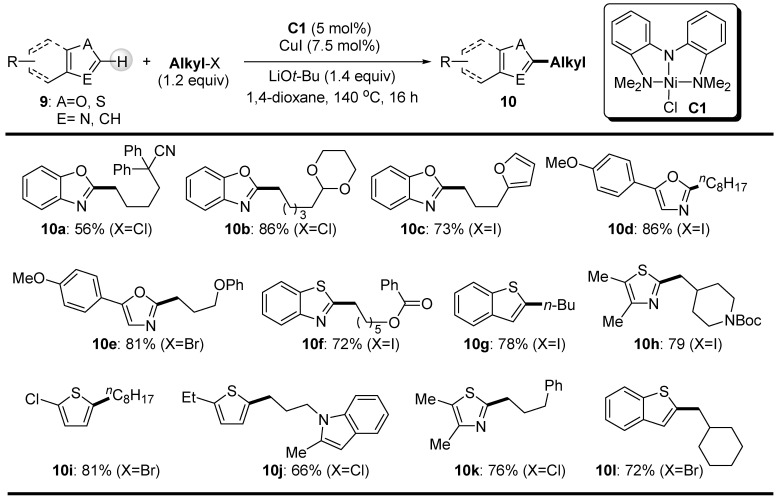
Well-defined Ni(II)-complex in the 2-C-alkylation of N, O and S-containing heteroaromatics.

In 2010, Hu et al. reported a versatile method for the alkylation of the C-H bond of N-, O- and S-containing heteroaromatics **9** with unactivated primary halides (chlorides, bromides, and iodides) (Scheme 5) [62]. This coupling reaction proceeded successfully through the cooperation of Ni(II)-complex and copper salt and showed excellent chemo- and regioselectivity in products **10** (Scheme 5). The alkylation reaction occurred exclusively at the 2-C position in both electron-poor and electron-rich heterocycles. Although the copper co-catalyst was not indispensable for the reaction, it contributed to the production of the alkylated heteroaromatics **10** in satisfactory yields. It was suggested that the copper facilitates the transmetalation of the anionic heterocyclic intermediate to the nickel center in the catalytic cycle. The Ni(II) complex **C1** bearing a tridentate *N*,*N*,*N*-ligand served as an efficient pre-catalyst that apparently degrades into active metal-nickel particles capable of coupling heteroarenes with alkyl halides.

**Scheme 6:**
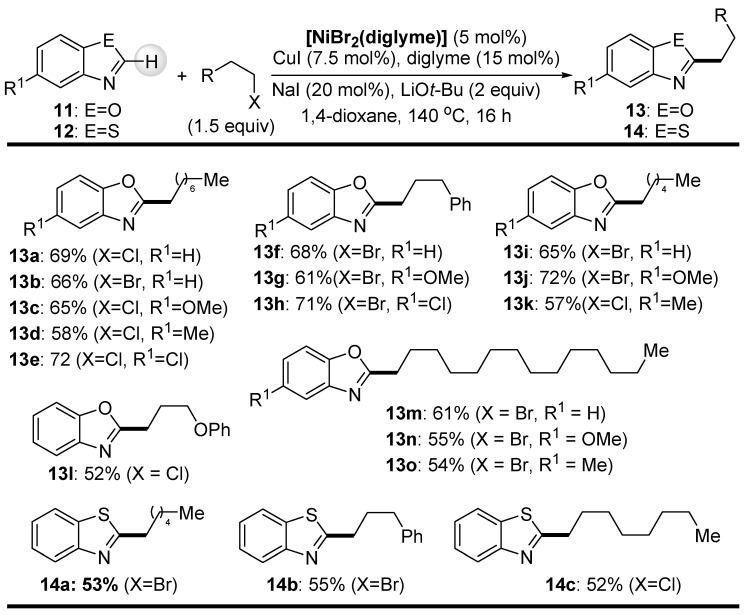
Cooperation of nickel and copper in the 2-C-alkylation of azaheteroaromatics.

Similar to Hu’s work, Ackermann and coworkers demonstrated that benzoxazoles **11** and benzothiazoles **12** can be 2-C-alkylated with unactivated primary alkyl halides, albeit using an inexpensive NiBr_2_/CuI catalyst system together with a catalytic amount of diglyme for efficient C-H bond cleavage (Scheme 6) [63]. The yields of the alkylated azaheteroaromatic products **13** and **14** were generally higher than the yields obtained by the Herbert group using well-defined Ni(II) complexes of pincer ligands in the same coupling reaction. Again, both primary alkyl bromides and chlorides can be used, but NaI must be added to achieve satisfactory yields. On the other hand, the secondary alkyl bromide 2-bromohexane proved to be almost unreactive under the applied reaction conditions.

**Scheme 7:**
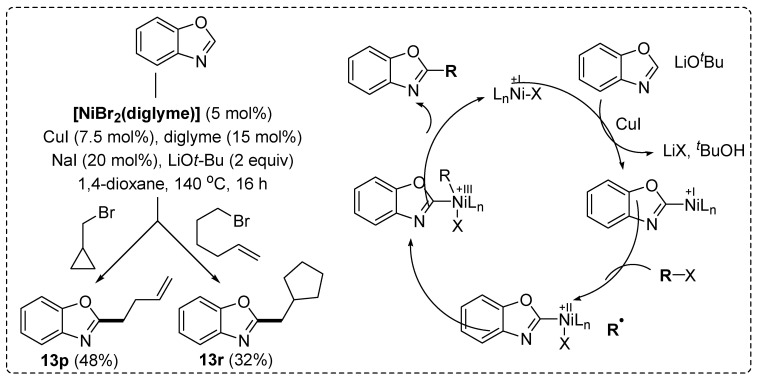
Mechanistic studies and possible pathway of nickel-catalyzed alkylation of fused azoles.

In order to gain an insight into the mode of action of the catalyst system for the alkylation reaction at hand, a few experiments were carried out (Scheme 7). The reactions of benzoxazole with cyclopropylmethyl bromide, giving **13p**, and with 6-bromohex-1-ene, giving the 5-exo-cyclization product **13r**, provided additional evidence for the formation of a radical intermediate involved in the catalytic cycle.

**Scheme 8:**
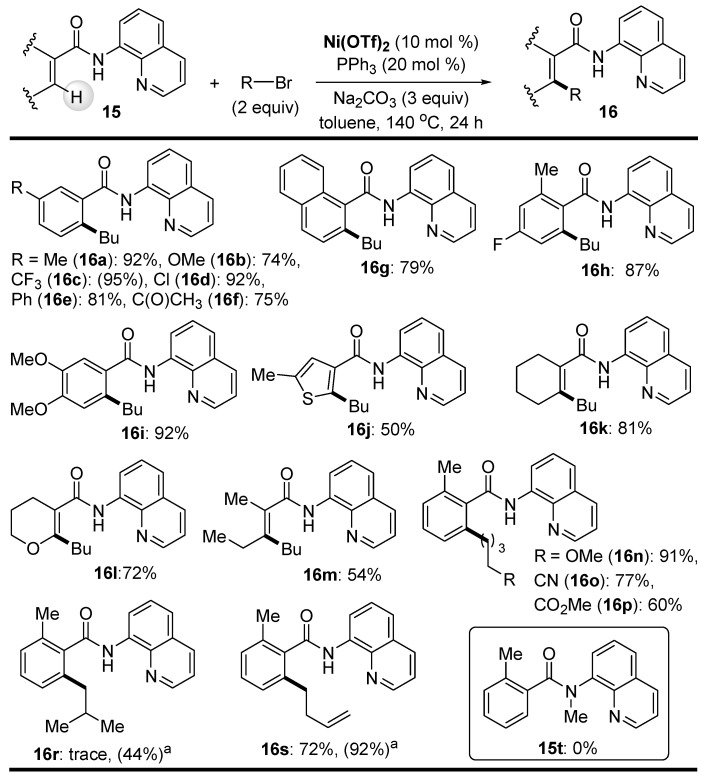
*N*,*N*-bidentate directing group in *ortho*-C-H bond alkylation of amides. ^a^ NaI (2 equiv.) added.

In 2013, Chatani and coworkers introduced an 8-quinolinylamine moiety as a powerful N,N-bidentate directing group in the Ru(II)-catalyzed *ortho*-arylation of aromatic amides [64]. This directing group was also found to be compatible with Ni(II) complexes, as the Ni(OTf)_2_/PPh_3_/Na_2_CO_3_ catalytic system efficiently performed alkylation of the *ortho*-C-H bond of benzamide derivatives and the *β*-C-H bond of *α*,*β*-unsaturated amides **15** (Scheme 8) [65]. Similarly, a thiophene ring of the heterocyclic amide was also alkylated (product **16j**). The cooperation of the nickel catalyst and the N,N-bidentate-directing group appeared to be very efficient in the present direct C-H bond alkylation, as a variety of unactivated primary alkyl bromides (both simple and functionalized) were successfully used as alkylating agents. Although the use of alkyl bromides gave high yields for most amide products **16**, the addition of NaI dramatically improved the isolated yields in some examples (**16r** and **16s**). This work was a great contribution to the approach to alkyl-substituted benzene derivatives, since the metal-catalyzed alkylation of a benzene ring is otherwise limited due to the unfavorable oxidative addition of alkyl halides and, moreover, the resulting alkyl metal intermediates tend to undergo a *β*-hydride elimination reaction [66]. Surprisingly, *α*,*β*-unsaturated amides lacking a substituent at the *α*-carbon were not reactive, thus limiting the reaction to trisubstituted alkene substrates giving the corresponding products **16k**–**m**. The authors also demonstrated that bidentate *N*,*N*-coordination by the 8-aminoquinolinyl moiety is essential for the reaction, as *N*-2-naphthylbenzamide and 8-quinolinyl benzoate do not give the desired alkylated products under the same reaction conditions. It has also been shown that the reaction does not proceed if no hydrogen is present on the nitrogen atom of the carboxamide group, as in substrate **15t**. 

**Scheme 9:**
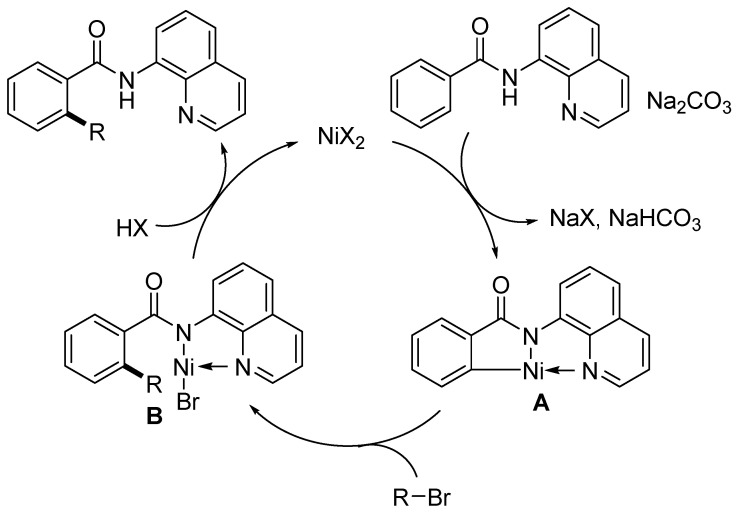
Simplified reaction pathway for the alkylation of the *ortho*-C-H bond through bidentate chelation assistance.

Based on additional studies (H/D exchange, competition experiments), the reaction pathway starting with amide coordination to the nickel atom, followed by cyclometallation giving the intermediate **A**, was proposed (Scheme 9). The oxidative addition of alkyl bromide, followed by reductive elimination, yields intermediate **B**, which, after protonation, gives the final alkylated amide product.

**Scheme 10:**
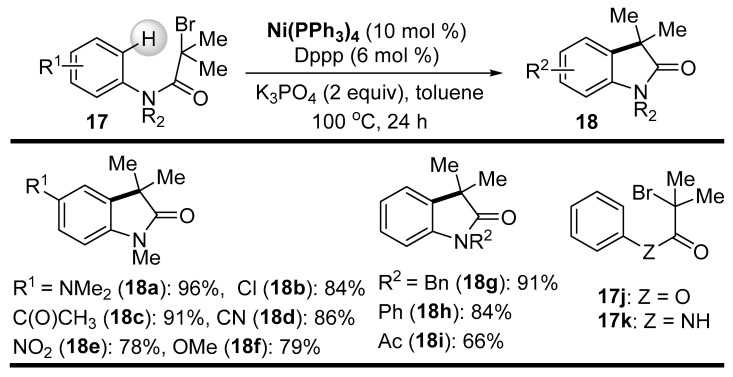
Intramolecular C-H alkylation with tertiary alkyl–Br bonds.

Lei and coworkers demonstrated a synthetically useful intramolecular alkylation of *ortho*-C-H bonds in a benzene ring of *N*-aryl *α*-bromoamide substrates **17** for the rapid preparation of indolone derivatives **18** [67]. The Ni(0) complex, together with the Dppp ligand, efficiently promoted this alkylation reaction, most likely via a radical process, as hypothesized on the basis of experiments with radical scavengers. Amide substrates **17** containing tertiary alkyl–Br bonds underwent this intramolecular cyclization smoothly and afforded the corresponding indolones **18** in about 80% yield, indicating an increased stability of the tertiary alkyl radicals (Scheme 10). 

**Scheme 11:**
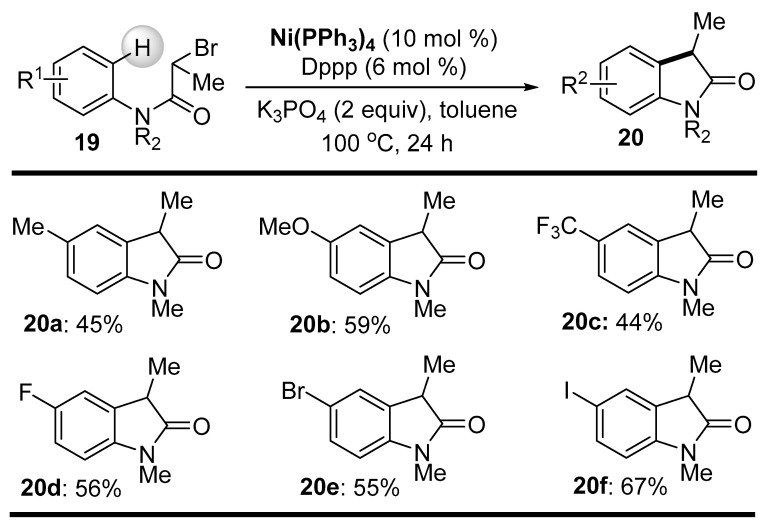
Intramolecular C-H alkylation with secondary alkyl–Br bonds.

Interestingly, the analogous ester substrate **17j** did not cyclize under these conditions, and, moreover, the amide **17k** with an NH group was also not reactive. However, the secondary alkyl–Br bonds in the amide substrates **19** were still reactive, but the yields of products **20** decreased significantly (average yield about 50%) (Scheme 11). 

**Scheme 12:**
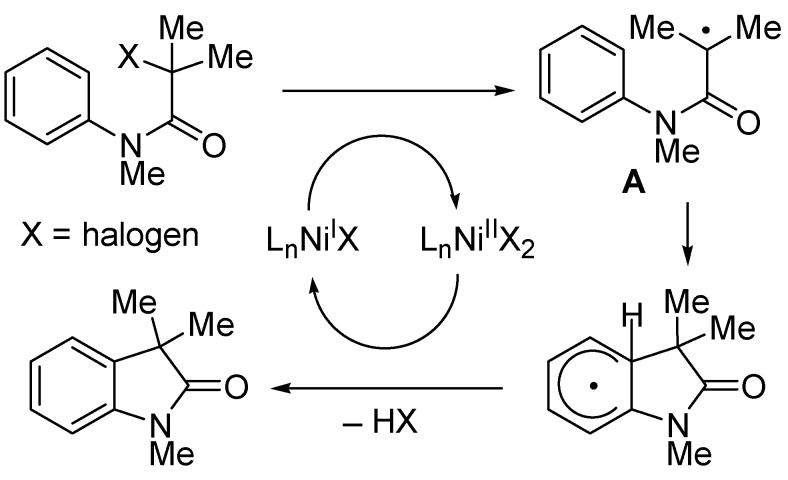
Proposed mechanism for intramolecular C-H alkylation of *N*-aryl *α*-bromoamides.

It is assumed that the above mentioned cyclization reaction proceeds via a catalytic Ni(I)/Ni(II) cycle. First, a Ni(0) complex reacts with an alkyl–X bond (X = halogen) to generate a catalytically active Ni(I) species, which then reacts with an alkyl halide to give a Ni(II) and alkyl radical species **A** (Scheme 12). Intramolecular radical addition to the aromatic ring and subsequent oxidation by Ni(II) with simultaneous deprotonation yields the final product and regenerates the Ni(I) species.

**Scheme 13:**
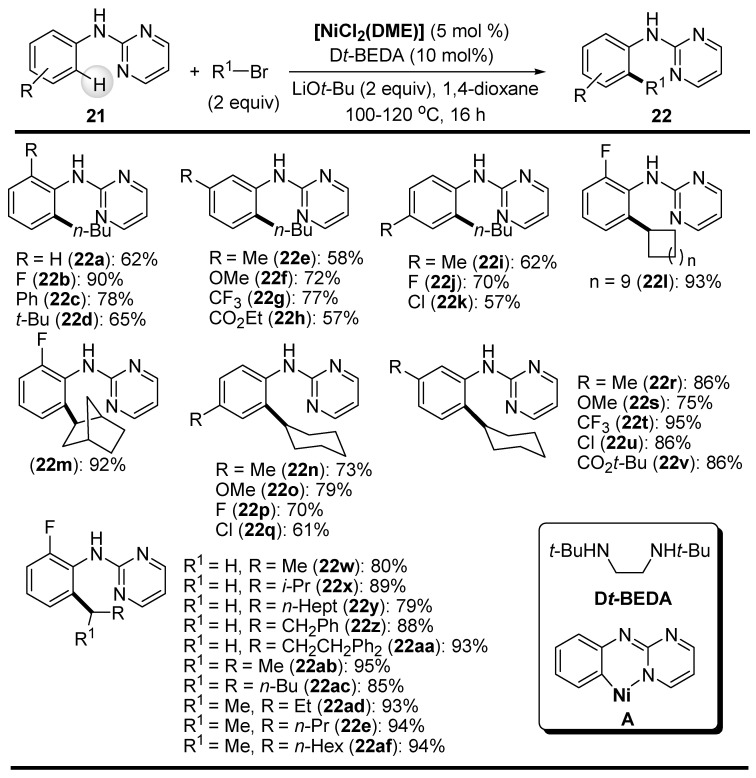
*Ortho*-C-H alkylation of *N*-pyrimidylanilines.

Another *ortho*-C-H bond alkylation, but using primary and secondary alkyl halides, was reported by Ackermann et al. [68]. They developed a protocol for the direct alkylation of anilines **21** bearing a 2-pyrimidyl moiety as a monodentate directing group on the nitrogen atom of the amino group. Interestingly, the reaction conditions introduced by Chatani et al. [66], who otherwise used the bidentate 8-quinolinylamine directing group (Scheme 8, *vide supra*), did not yield the desired *ortho*-alkylated aniline products for the nickel-catalyzed alkylation shown in Scheme 13. It was hypothesized that a vicinal diamine ligand D*t*-BEDA plays an important role in the catalytic process mediated by the Ni(II) salt to achieve high yields of alkylation products **22**. This could be due to the ligand-promoted formation of normally thermodynamically less stable six-membered metallacycle intermediates, such as **A**, in the catalytic cycle. A broad range of functionalized primary and secondary alkyl bromides can be coupled with *ortho*-, *meta*-, and *para*-substituted anilines with different functionalities in a highly chemo- and regioselective manner. 

**Scheme 14:**
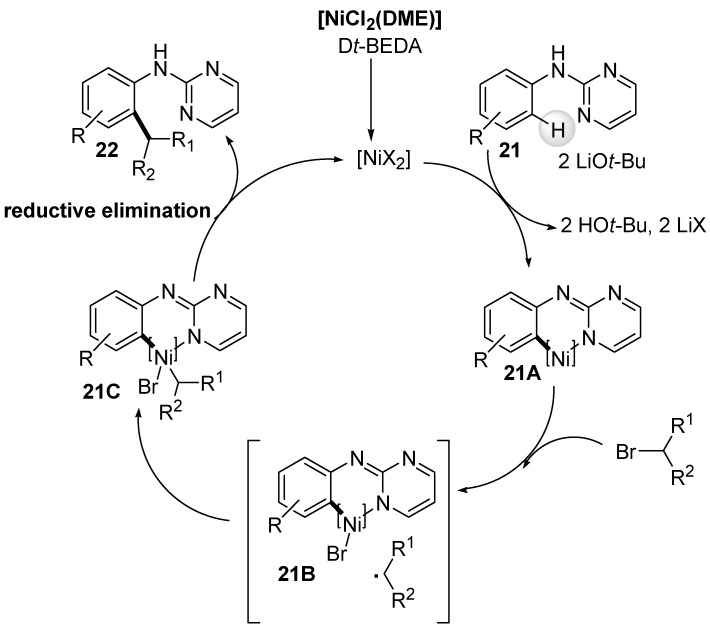
Proposed mechanism for Ni-catalyzed C–H alkylation of 2-pyrimidinyl anilines with alkyl halides.

Based on the mechanistic studies performed, a plausible mechanism was proposed (Scheme 14) [68]. The initial [NiCl_2_(DME)] precatalyst reacts with D*t*-BEDA ligand to form an active [NiX_2_] catalyst. This active catalyst initiates the catalytic cycle by reacting with 2-pyrimidinyl aniline **21** in the presence of two equivalents of LiO*t*-Bu as a base. Deprotonation of the aniline NH, coordination with [Ni], followed by C-H bond cleavage affords a six-membered cyclometalated complex **21A**. Single electron transfer between the alkyl halide and the cyclometalated complex **21A** gives intermediate **21B** and an alkyl radical. The subsequent reaction affords cyclometalated complex **21C**, which, upon reductive eliminations, gives rise to the alkylated 2-pyrimidinyl aniline **22** with simultaneous regeneration of the active [NiX_2_] catalyst (Scheme 14).

**Scheme 15:**
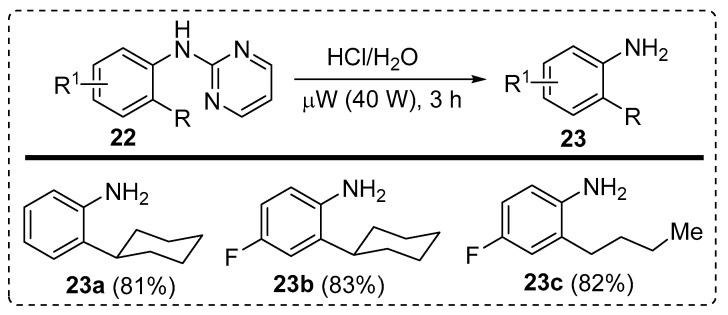
Easy removal of the pyrimidyl directing group.

The synthetic utility of pyrimidinyl-assisted *ortho*-C-H alkylation of the benzene ring of anilines was demonstrated by a simple removal of the 2-pyrimidinyl directing group (Scheme 15) [68]. Heating of the alkylation products **22** in aqueous HCl under microwave conditions afforded the corresponding *ortho*-alkylated anilines **23** with a free amino group that can be used for further synthetic manipulations (Scheme 15).

**Scheme 16:**
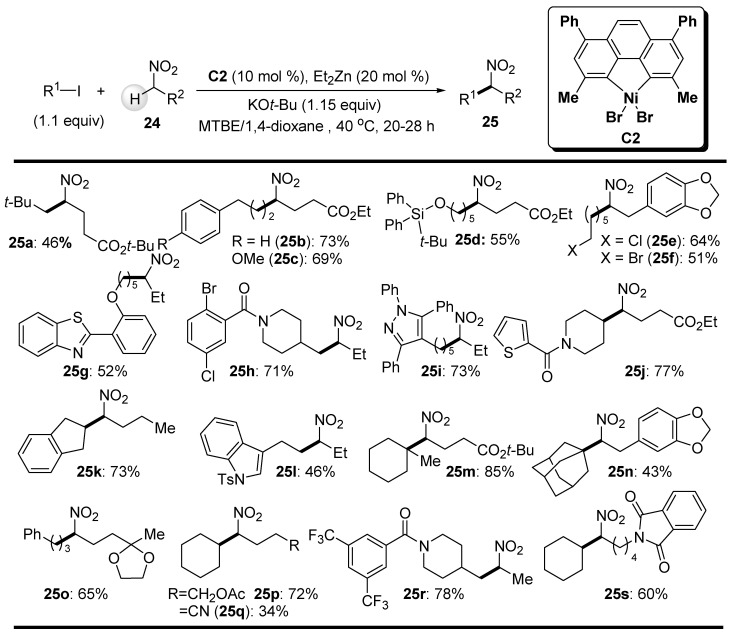
C-H bond alkylation of nitroalkanes with unactivated alkyl iodides.

In 2017, Watson and coworkers reported the general catalytic C-H bond alkylation of nitroalkanes with unactivated alkyl halides using the single-component nickel catalyst **C2** prepared from a Ni(II) precursor and bathocuproine ligand (Scheme 16) [69]. Importantly, this catalyst, together with a catalytic amount of ZnEt_2_ as a reducing agent, was active at a temperature of 40 °C and gave the branched nitroalkanes in moderate-to-good yields. The generality of this protocol lies in the use of primary, secondary (cyclic and acyclic) and tertiary alkyl iodides as alkylating agents with different functionalities (e.g., heteroaryl, silyl, carboxamide) in the preparation of branched nitroalkanes **25**. In addition, different functional groups (e.g., ester, nitrile, imide) are well-tolerated on the nitroalkane substrates **24**. Interestingly, alkyl chlorides and bromides were not affected under the applied reaction conditions, as demonstrated by the successful preparation of bromo- and chloronitroalkanes, **25e** and **25f**. Although a nitroalkane with a ketone group did not give the desired alkylated product, its acetal protection enabled a good yield of product **25o** (Scheme 16).

**Scheme 17:**
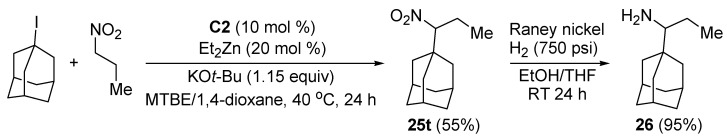
Synthesis of adapromine by alkylation of nitroalkanes.

The synthetic utility of this alkylation reaction was demonstrated by the preparation of a pharmaceutically relevant compound adapromine (**26**) (Scheme 17). First, 1-nitropropane was alkylated with 1-adamantyl iodide according to an optimized reaction protocol. The resulting product **25t** was then hydrogenated under Raney nickel conditions, giving the target compound **26** in an overall yield of 52% (Scheme 17).

**Scheme 18:**
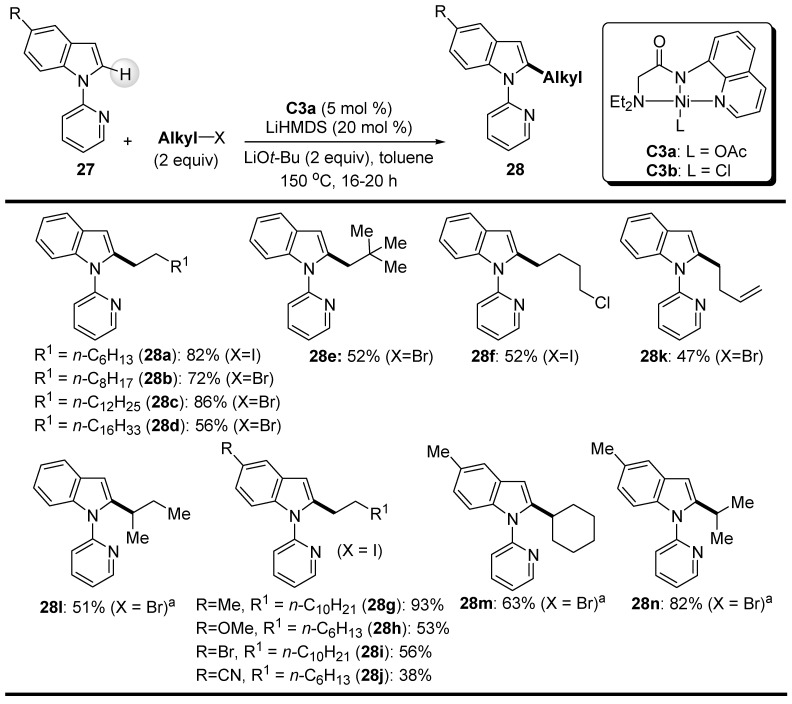
(Quinolinyl)amido-Ni(II) catalyst in the C-2 alkylation of indoles. ^a^ KI (2 equiv.) was added.

Punji et al. developed a synthetic method for the direct 2-C-H alkylation of *N*-(2-pyridyl)indoles **27** with alkyl halides in the presence of the Ni(II) catalyst **C3** containing a pincer-type ligand (Scheme 18) [70]. Of the two catalysts tested, the acetate **C3a** was more efficient than the chloride **C3b** in coupling of 1-iodooctane with *N*-pyridylindole, giving the 2-alkylated indole **28a** in a yield of 82%; the catalyst **C3b** gave 53% of the same product. Primary and secondary iodides and bromides were used as coupling partners, and the reaction had to be carried out at a high temperature of 150 °C. When secondary bromides were used as alkylating agents, the addition of two equivalents of KI was required to obtain satisfactory yields of the corresponding alkylated products **28l**–**n**. Importantly, a 2-pyridyl directing group on the nitrogen atom of the indole substrates was crucial to obtain regioselectively 2-alkylated indole derivatives **28** (Scheme 18).

**Scheme 19:**
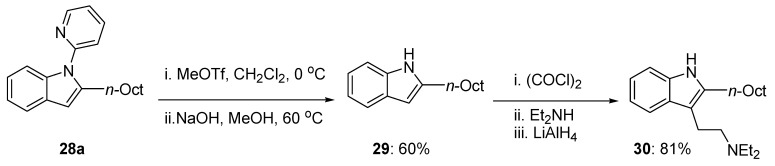
Synthesis of tryptamine alkaloid.

The synthetic value of the present coupling method was justified by the synthesis of the tryptamine alkaloid **30** (Scheme 19) [70]. A simple removal of the 2-pyridyl group in the starting indole **28a** with MeOTf afforded the corresponding free NH-indole **29** in 60% yield. Further functionalization at the 3-C of indole **29** gave the desired tryptamine derivative in 81% yield (Scheme 19) [70]. 

**Scheme 20:**
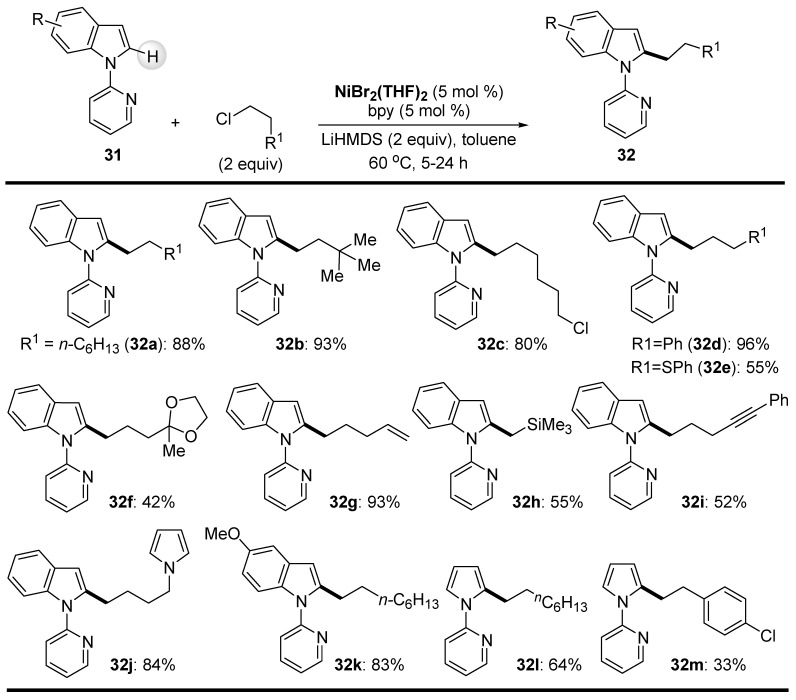
Directing group-assisted 2-C-H alkylation of indoles and pyrroles with primary chlorides.

As an improvement of their aforementioned work, Punji et al. described a milder (60 °C) yet efficient coupling of alkyl halides and 2-C-H bond not only of *N*-(2-pyridyl)indoles but also of *N*-(2-pyridyl)pyrroles **31** (Scheme 20) [71]. It was shown that the use of the nickel catalyst system NiBr_2_·(THF)_2_/Bpy, together with the strong base LiHMDS, gives the corresponding 2-alkylated indole and pyrrole derivatives **32a**–**k** and **32l**–**m**, respectively. Milder bases, such as KO*t*-Bu or K_2_CO_3_, gave only traces of the coupling product or were ineffective. Apparently, the cooperation of Ni(II) species, the ligand Bpy and a strong base is responsible for the successful coupling of indoles with alkyl halides at lower temperatures of 60 °C. Remarkably, less expensive (compared to analogous bromides or iodides) and readily available primary alkyl chlorides were used for this catalytic reaction, which gave good-to-excellent yields (up to 96%) of the 2-alkylated heteroaromatic products **32**. While primary and secondary alkyl halides reacted smoothly under the applied conditions, coupling with *tert*-butyl chloride or 1-chloroadamantane as sterically demanding reactants was not observed. The reaction protocol tolerated a wide range of functional groups (e.g., acetal, thioether, silyl, heteroaryl, alkenyl and alkynyl) in the primary alkyl chlorides, but the electrophiles containing a -CO_2_R, -CN or -NO_2_ group decomposed under the applied reaction conditions. Surprisingly, 1,6-dichlorohexane reacted very efficiently, with only one (*sp*^3^)C-Cl under optimized conditions, giving the product **32c** in high yield (80%) (Scheme 20). In addition, some mechanistic studies were carried out, which revealed that a single-electron transfer process involving the Ni(I) and Ni(III) species could occur during the catalytic cycle.

**Scheme 21:**
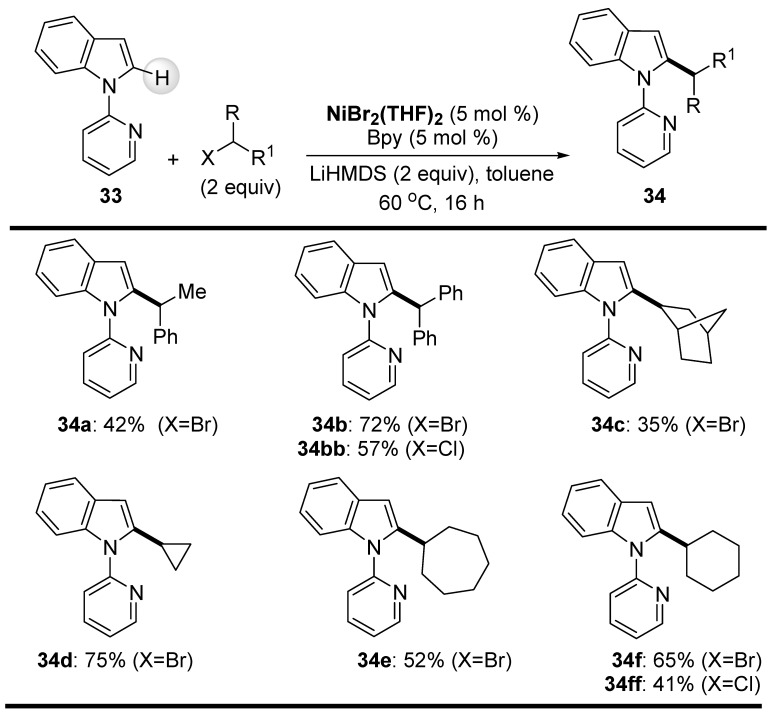
Directing group-assisted 2-C-H alkylation of indoles with secondary halides.

The optimized reaction conditions were also applied to the coupling reaction of secondary alkyl halides with the 2-C-H bond of *N*-(2-pyridyl)indole (**33**) (Scheme 21) [71]. Phenyl-substituted acyclic chlorides and bromides afforded products **34a** and **34b** in moderate-to-good yields, whereas coupling of 2-chlorobutane and 2-chloropentane with the same indole **33** afforded only trace amounts of target products. Cyclic secondary alkyl halides coupled smoothly with indole **33** and afforded 2-alkylated indoles **34d**–**34f** in satisfactory yields. On the other hand, the reactivity of 2-bromobicyclo [2.2.1]heptane was slightly lower under the optimized conditions (35% of product **34c**), probably due to the increased steric effect of this particular electrophile (Scheme 21).

**Scheme 22:**
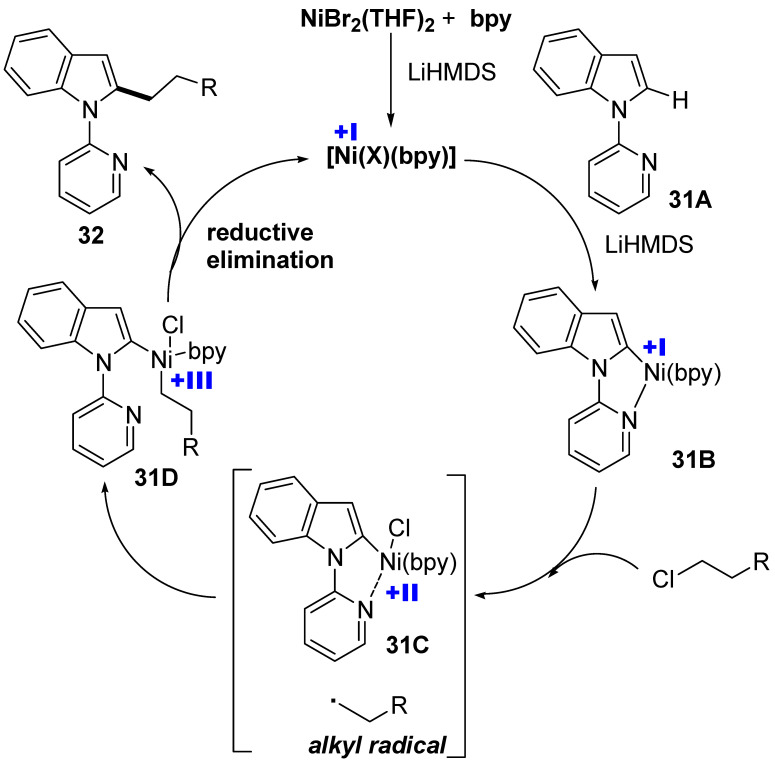
Proposed mechanism for Ni-catalyzed C-H alkylation of 2-pyridinyl indoles with alkyl halides.

After a series of mechanistic investigations, a plausible catalytic pathway was postulated (Scheme 22) [71]. The initial Ni(II) in NiBr_2_(THF)_2_ was reduced to an active Ni(I) species, [Ni(X)(bpy)], in the presence of LiHMDS, as supported by EPR and XPS studies. Additionally, 2-Pyridinyl indole **31A** then reacted with the resulting low-valent Ni(I) in the presence of LiHMDS to produce cyclometalated Ni-complex **31B**. As a rate-determining step, the alkyl chloride underwent a radical formation triggered by the Ni-complex **31B**, giving rise to the Ni species **31C** and the alkyl radical. The decoordination between the Lewis basic N atom in the pyridinyl moiety and the Lewis acidic Ni, followed by the formation of the Ni–C bond between Ni and the radical-bearing C in the alkyl radicals, gave the Ni intermediate 31D. Final reductive elimination furnished the alkylated 2-pyridinyl indole **32** with concomitant regeneration of the active low-valent Ni(I) catalyst (Scheme 22) [71].

**Scheme 23:**
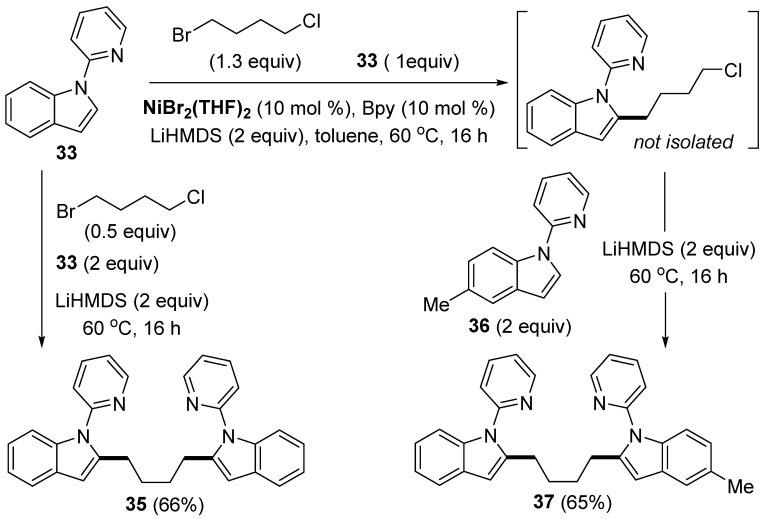
One-pot synthesis of bis(indolyl)alkane scaffolds.

The authors also investigated this coupling strategy in the preparation of symmetrical and, in particular, unsymmetrical bis(indolyl)alkane scaffolds using a one-pot synthetic strategy (Scheme 23). Treatment of the starting indole **33** (2 equiv.) with 1-bromo-4-chlorobutane (0.5 equiv.) gave a symmetrical bis(indolyl)butane **35**, while an unsymmetrical analog **37** was prepared by a sequential reaction of 1-bromo-4-chlorobutane (1.3 equiv.) with indoles **33** (1 equiv.) and **36** (2 equiv.); additionally, the monoindolation product was not isolated (Scheme 23).

**Scheme 24:**
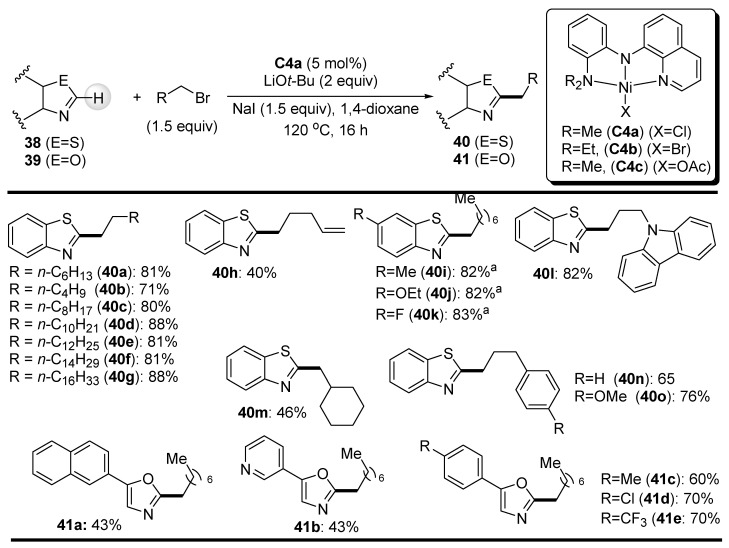
Nickel(II) complexes of quinoline-based *N*,*N*,*N*-ligands in alkylation of benzothiazoles and benzoxazoles. ^a^ Using alkyl iodide instead of bromide without addition of NaI.

Punji et al. extended the scope of direct 2-C-alkylation of fused azaheteroarenes (see Scheme 24) by using well-defined Ni(II) complexes of tridentate *N,N,N*-pincer ligands. Thus, the complexes **C4** of quinoline-based ligand were investigated in the catalytic C-H bond alkylation of benzothiazoles **38** and oxazoles **39** with primary alkyl halides (Scheme 22) [72]. Complex **C4a** showed the best performance in the model reaction of 1-iodooctane with benzothiazole and proved to be a very robust catalyst in the present coupling reaction, as it can be reused five times without a significant decrease in its activity. For example, the yield of product **40a** in the reaction of benzothiazole with 1-iodooctane decreased from 92% to 85% under optimized conditions when moving from the first to the fifth cycle. In particular, the extent of the alkylation reaction with readily available primary alkyl bromides was investigated, but in order to obtain satisfactory yields of products **40** and **41**, NaI (1.5 equiv.) was added to the reaction mixture (Scheme 24).

**Scheme 25:**
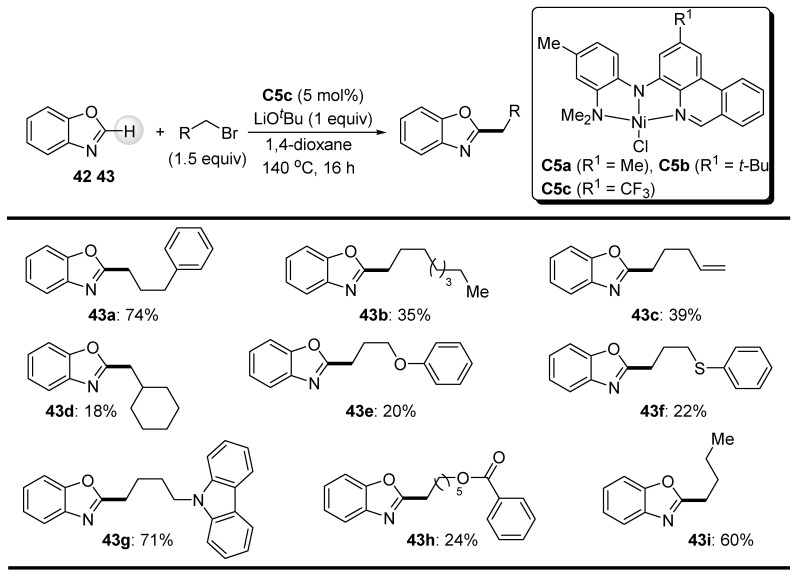
Nickel(II) complexes of phenanthridine-based *N*,*N*,*N*-ligands in alkylation of benzoxazoles.

As an exploration of the direct alkylation method mentioned above (Punji’s work), nickel complexes **C5** ligated with *NNN*-tridentate but phenanthridine-based ligands proved to be satisfactorily efficient catalysts in the functionalization of benzoxazole (**42**) with unactivated primary alkyl bromides in a highly site-selective manner, as reported by Herbert et al. (Scheme 25) [73]. Of the nickel complexes tested, complex **C5c** showed the best catalytic performance in this coupling reaction. Although the yields of 2-alkylated benzoxazole derivatives **43** were generally not high, the examples shown in Scheme 25 represent a significant contribution to nickel-promoted functionalization of C-H bonds, particularly because well-defined Ni(II) complexes were used and pincer-like ligands can efficiently stabilize a catalytically active nickel center.

**Scheme 26:**
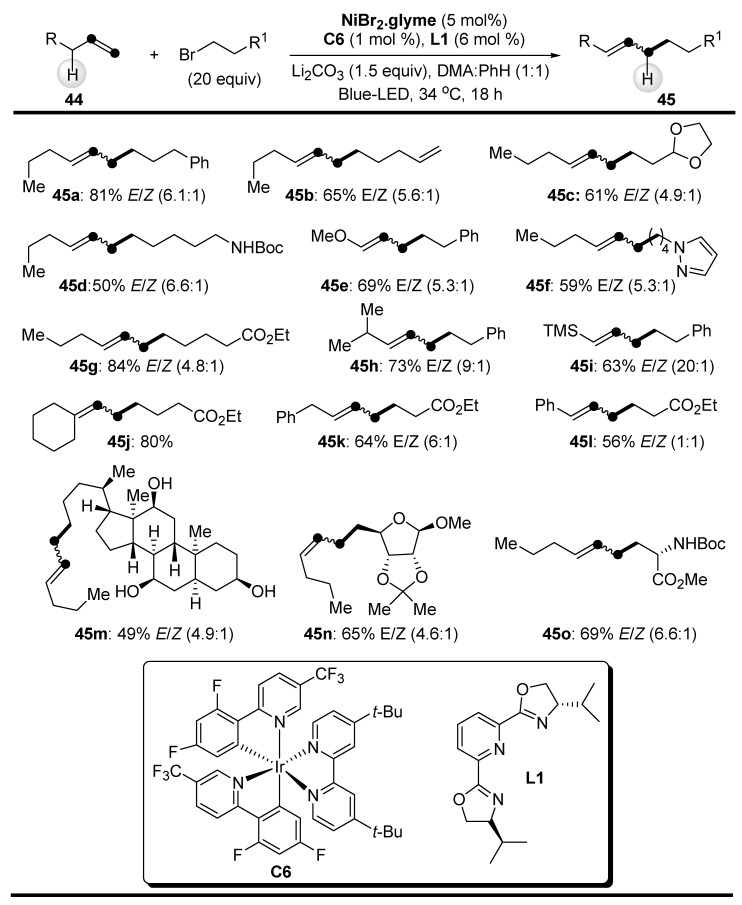
Nickel catalysis in combination with iridium photocatalysis in coupling of α-olefins with primary alkyl bromides.

Recently, Martin et al. discovered that unactivated *α*-olefins **44** and unactivated alkyl bromides elegantly couple under light-induced nickel catalysis to readily form a C(sp^3^)–C(sp^3^) linkage between the *α*-carbon atom of olefins and an alkyl moiety of alkyl halides by simultaneous hydrogen atom transfer at the allylic C(sp^3^)–H bond [74]. This coupling protocol proved to be widely applicable, as primary and secondary alkyl bromides with different functionalities (e.g., acetal, ester, alcohol, carbamate, sulfonamide, alkenyl*,* alkynyl) can be used to chemoselectively obtain moderate-to-good yields of internal olefin products **45** (Scheme 26). The catalyst system NiBr_2_·glyme/**L1**/Li_2_CO_3_, used together with the photoactive iridium complex **C6**, allowed a smooth alkylation reaction with primary alkyl bromides at 34 °C, obtaining products **45** in good yields (Scheme 24). Of particular interest is the use of pyrazole-containing primary alkyl bromide, which does not interfere with the formation of the alkylated product **45f**, indicating that the pyrazole ring does not compete with the ligand **L1** for binding to the nickel center in the catalytic cycle. It is worth noting that this protocol was also suitable for alkyl bromides containing amino acid, saccharide and steroid fragments (with free alcohol groups), resulting in the products **45m**–**o** (Scheme 26).

**Scheme 27:**
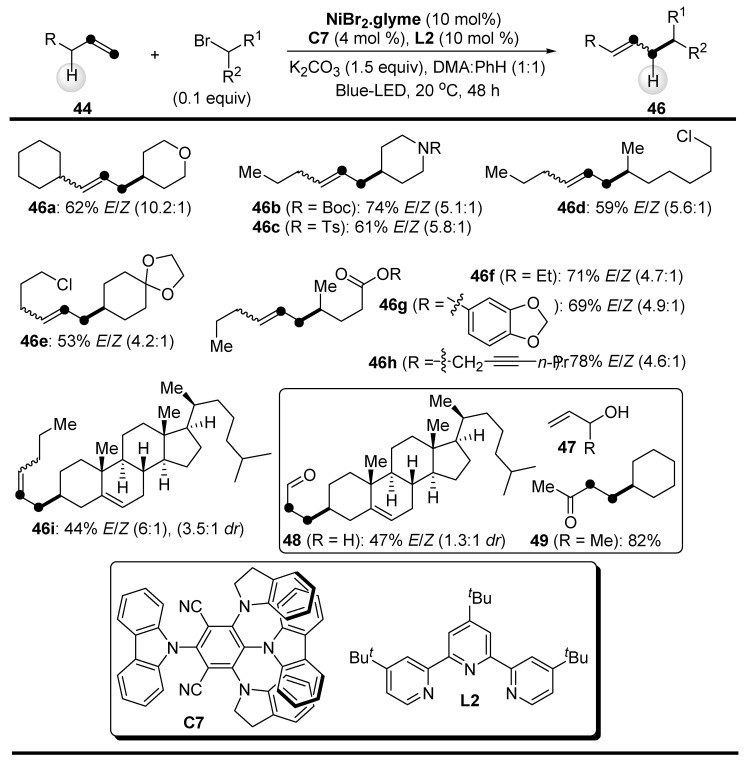
Nickel catalysis in combination with metal-free photocatalysis in coupling of α-olefins with secondary alkyl bromides.

On the other hand, a protocol based on the NiBr_2_·glyme/**L2**/K_2_CO_3_ catalytic system, together with a metal-free photoredox catalyst **C7**, was more suitable for alkylation with secondary alkyl bromides (acyclic and cyclic) in regard to giving olefinic products **46** (Scheme 27). Remarkably, aldehyde **48** and ketone **49** were obtained in good yields when the parent alkyl bromides were coupled with allylic alcohol **47** as an olefinic coupling partner under the same reaction conditions (Scheme 27). Although mixtures of *E*/*Z*-isomeric olefin products were isolated in the present coupling reactions, the developed protocol allowed the synthesis of densely functionalized compounds from simple starting materials.

### 2.3. Miscellaneous C-H Bond Alkylations

**Scheme 28:**
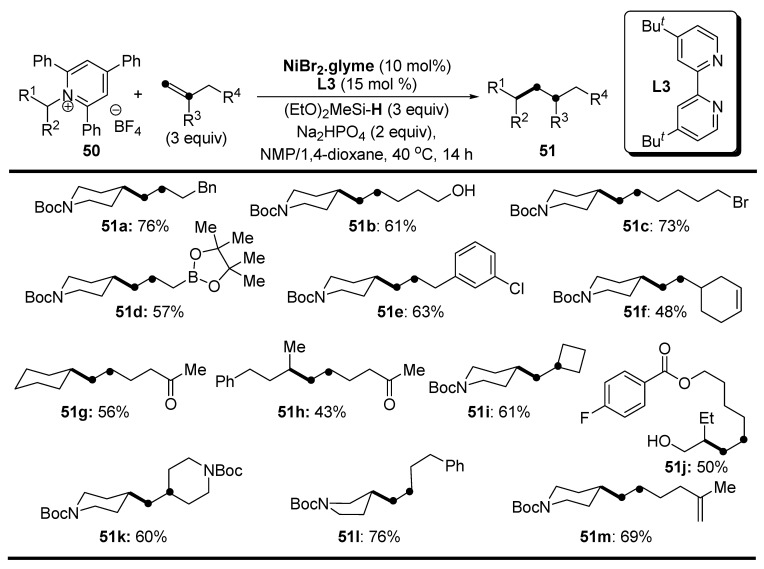
Deaminative alkylation of unactivated α-olefins.

Martin et al. presented an interesting deaminative alkylation of unactivated olefins with pyridinium salts **50** (which can be easily prepared from the corresponding amines and pyrylium salts) via their (*sp*^3^)C–N bond cleavage (Scheme 28) [75]. This unique coupling reaction proceeds as a 1,2-hydroalkylation of the C=C double bond of the olefins to generate a new (*sp*^3^)C–(*sp*^3^)C bond in the products **51** or **52** in the presence of catalytic amounts of Ni(II) salt/ligand, a hydride source ((EtO)_2_MeSi–H or (EtO)_3_Si–H) and Na_2_HPO_4_ as base (Scheme 28). It was found that, for the alkylation of terminal olefins, the NiBr_2_·glyme complex, together with the bipyridine ligand **L3**, is the privileged catalyst system that operates successfully at 40 °C with excellent *anti*-Markovnikov selectivity (Scheme 26). It is worth mentioning that only the mixture of NMP and 1,4-dioxane as a solvent system gave the best reaction outcome, as a single solvent gave a lower yield (Scheme 28).

**Scheme 29:**
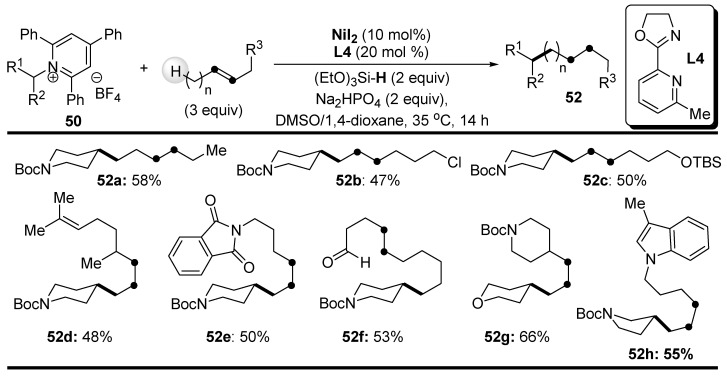
Deaminative alkylation of unactivated internal olefins.

On the other hand, the stabilization of the catalytically active nickel center (originating from NiI_2_) in the deaminative alkylation of internal olefins was achieved by the oxazoline-pyridine ligand **L4** (Scheme 29). Although internal olefins were used as substrates, (*sp*^3^)–C alkylation occurred at a distant primary (*sp*^3^)C-H site, which gave products **52** regardless of the position of the double bond. In the alkylation of internal olefins, a DMSO/1,4-dioxane solvent system was used, while the reaction took place at a lower temperature of 35 °C (Scheme 29).

**Scheme 30:**
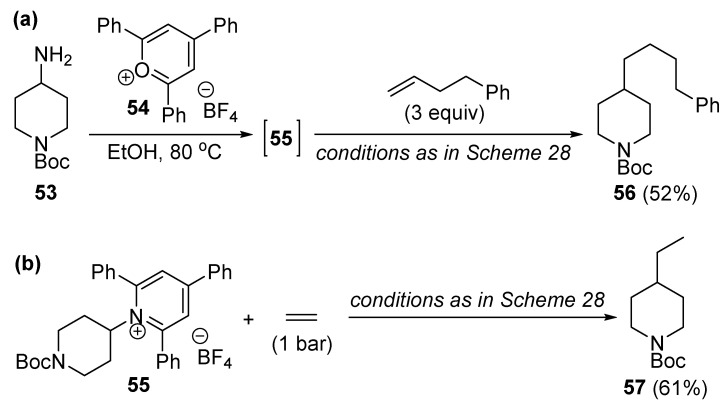
(**a**) Deaminative one-pot alkylation without isolation of pyridinium salt. (**b**) Use of ethylene as substrate.

As far as synthetic applications are concerned, the authors have shown (albeit in only one case) that the deaminative alkylation of olefins can also be achieved with the in situ formed pyridinium salt **55** from the parent piperidinyl amine **53** and pyrylium salt **54** using a one-pot synthetic strategy (Scheme 30a). It is worth noting that ethylene, as an industrially important chemical, can also be successfully used as a coupling partner under atmospheric pressure to produce ethylated molecular scaffolds (product **57**, Scheme 30b).

**Scheme 31:**
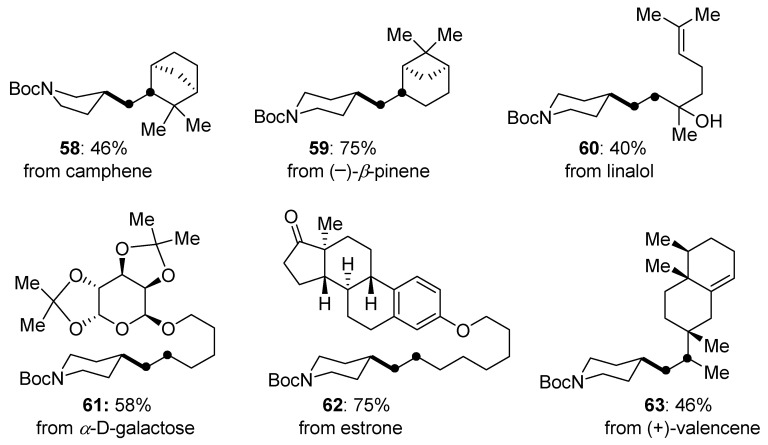
Natural product derivatives obtained by deaminative alkylation in late-stage functionalization.

Finally, it was shown in several cases that the developed protocol can be applied for the late-stage selective functionalization of substrates derived from natural products, e.g., camphene, (−)-*β*-pinene, linalol, D-galactose, estrone, and (+)-valencene (products **58**–**63**, Scheme 31).

### 2.4. Asymmetric C-H Bond Alkylation

**Scheme 32:**
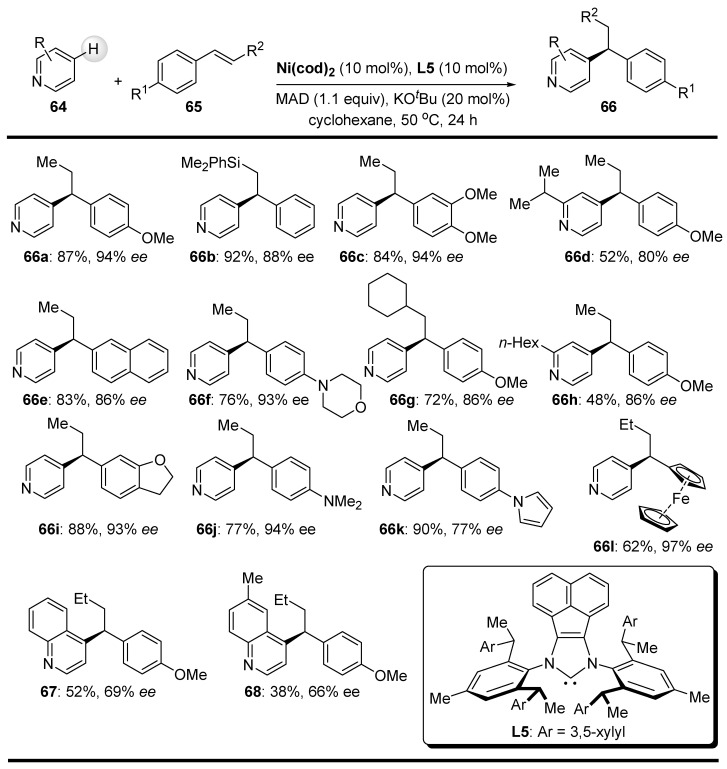
Asymmetric addition of the pyridine *ortho*-C-H bond to styrenes.

Recently, Shi’s group published the then first enantioselective C-H bond alkylation of pyridines **64** with *β*-substituted styrenes **65**, which was realized using a bimetallic nickel-aluminum catalyst system with an *N*-heterocyclic ligand **L5** (Scheme 32) [76]. The reaction proceeded formally as an asymmetric addition of a *para*-C-H bond of pyridine **64** across the C=C double bond of the alkene, leading to 1-aryl-1-pyridylalkanes **66**. Enantiocontrol was provided by a *C*_2_-symmetric chiral acenaphthene-type NHC ligand **L5** bearing bulky 3,5-Xylyl groups. It is noteworthy that the present activation of the pyridyl C-H bond was not possible without the Lewis acid methylaluminium bis(2,6-di-*tert*-butyl-4-methylphenoxide) (MAD) or with smaller Lewis acids, such as AlMe_3_, AlEt_3_ or Al(*^i^*Bu)_3_. It is suggested that the complete *para*-regioselectivity in pyridine substrates is due to the steric effect of MAD in a Lewis acid–base-MAD–pyridine adduct, which prevents the functionalization of the pyridyl *ortho*-C-H bond. As shown in Scheme 30, a wide range of substituted styrene and pyridine coupling partners were successfully utilized in this catalytic reaction, leading to 1,1-diarylalkanes with pyridyl moieties in high yields and good-to-excellent enantioselectivities at a temperature of 50 °C. Interestingly, a ferrocene-containing alkene also reacted smoothly under slightly modified conditions (3 equiv. of alkene, 60 °C, 48 h), affording the alkylated pyridine derivative **66l** with an exceptionally high enantioselectivity (97% *ee*). In addition, quinoline substrates also afforded the desired 4-C-H alkylated products **67** and **68**, albeit in moderate yield, with good enantioselectivity (~65% *ee*) (Scheme 32).

**Scheme 33:**
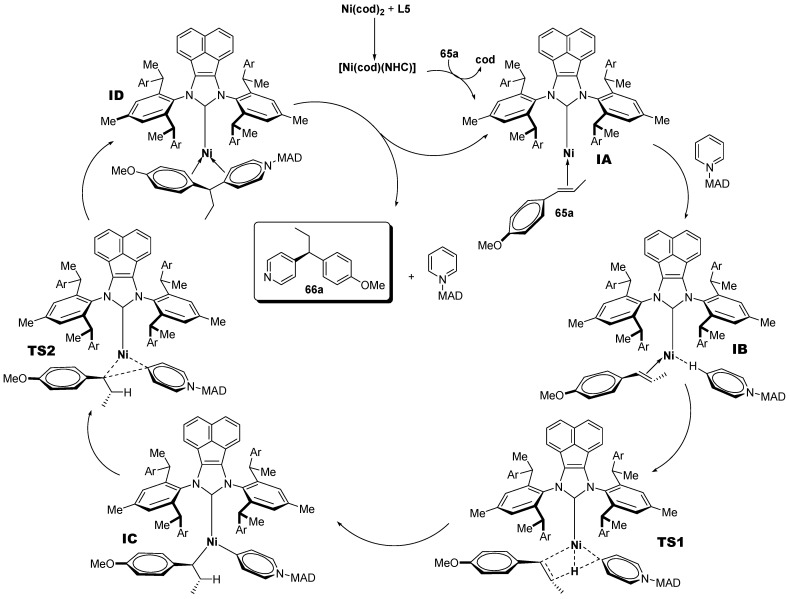
Proposed mechanism for Ni-catalyzed C-H alkylation of pyridines with styrenes.

Mechanistic investigations were carried out for the Ni-catalyzed C-H alkylation of pyridines with styrenes [76]. Based on previous findings and calculated Gibbs energy profiles, the reaction mechanism is believed to begin with substrate coordination, proceeds through ligand to ligand hydrogen transfer (LLHT), before ending with reductive elimination. A plausible mechanism was postulated on the basis of energy calculations (Scheme 31). The mechanism given below applies to the energetically more favorable S isomers of the intermediates and transition states in the energy profile [76]. The Ni precatalyst, Ni(cod)_2_, undergoes NHC ligand **L5** exchange with cod to give an active Ni catalyst, [Ni(cod)(NHC)]. The active Ni catalyst then coordinates with the trans styrene **65a** to form intermediate **IA**. The intermediate **IA** then coordinates with the Lewis adduct formed by the reaction between the Lewis basic pyridine **64** and the Lewis acidic MAD (Scheme 32, *vide supra*) to give intermediate **IB**. The LLHT then takes place through transition state **TS1**, which gives rise to the intermediate **IC**. The intermediate **IC** undergoes reductive elimination through transition state **TS2** to form the intermediate **ID**, which then releases the pyridine alkylated product **66a** by ligand exchange along with pyridine-MAD Lewis adduct, simultaneously regenerating the **IA** (Scheme 33).

## 3. Conclusions

Ni-catalyzed alkylation of C-H bonds is an attractive strategy for the direct functionalization of inert C-H bonds of various substrates. One advantage is the use of Ni as a first-row transition metal, which occurs in larger quantities in the earth’s crust and is more environmentally friendly than the second-row transition metals. Another advantage is the possibility of incorporating alkyl groups into valuable molecules, which allows access to natural product frameworks with differently functionalized alkyl groups. This review focuses on the Ni-catalyzed C-H bond alkylation reactions of various carbocyclic and heterocyclic substrates with alkyl halides and olefins. The alkylation reactions covered include, for example, intramolecular alkylation for the rapid construction of indolone scaffolds. The Ni-catalyzed C-H bond alkylation of aromatic and unsaturated amides with alkyl halides and of nitroalkanes with unactivated olefins is also covered. The regioselectivity of C-H bond alkylation in unactivated substrates was, in many cases, ensured by chelation assistance with monodentate pyridyl and pyrimidyl or bidentate quinolinylamine directing groups. The unique enantioselective alkylation of pyridines with alkenes paves the path for future Ni-catalyzed asymmetric alkylations.

## Data Availability

No new data were created or analyzed in this study. Data sharing is not applicable to this article.

## Abbreviations

Ac	Acyl
Ar	Aryl
Bn	Benzyl
Boc	*tert*-Butyloxycarbonyl
Bpy	2,2-Bipyridine
Bu	Butyl
Cbz	Benzyloxycarbonyl
Cod	1,5-Cyclooctadiene
Cp	Cyclopentadienyl
Cy	Cyclohexyl
Cyp	Cyclopentyl
DCM	Dichloromethane
diglyme	bis(2-methoxyethyl) ether
DMA	Dimethylacetamide
DME	Dimethoxyethane
DMSO	Dimethylsulfoxide
dppp	1,3-Bis(diphenylphosphino)propane
D*t*BEDA	*N*^1^,*N*^2^-di-*tert*-butylethane-1,2-diamine
*ee*	enantiomeric excess
equiv	Equivalent
Et	Ethyl
glyme	Dimethoxyethane
Hept	Heptyl
Hex	Hexyl
HMDS	Hexamethyldisilazide
*^i^*Pr	*iso*-Propyl
L	Ligand
LED	Light-emitting diode
Me	Methyl
MTBE	Methyl *tert*-butyl ether
NHC	*N*-Heterocyclic carbine
NMP	*N*-Methyl-2-pyrrolidone
Ph	Phenyl
Pr	Propyl
RT	Room Temperature
TBME	*tert*-Butyl methyl ether
TBS	*tert*-Butyldimethylsilyl
Tf	Triflyl
THF	Tetrahydrofuran
TM	Transition meta
TMS	Trimethylsilyl
Xantphos	(9,9-Dimethyl-9*H*-xanthene-4,5-diyl)bis(diphenylphosphane)
Xylyl	Dimethylphenyl
μW	Microwave
